# Attitudes of Graduating Medical and Nursing Students Toward Older Persons in Ghana

**DOI:** 10.1093/geroni/igaa057.701

**Published:** 2020-12-16

**Authors:** Grace Karikari, Huber Lesa, David Lohrmann, Margaret Adamek, Karo Omodior

**Affiliations:** 1 Indiana University School of Public Health, Grand Forks, North Dakota, United States; 2 Indiana University Bloomington, Bloomington, Indiana, United States; 3 Indiana University Purdue University Indianapolis, Indianapolis, Indiana, United States

## Abstract

Purpose: Differences in attitudes between graduating medical and nursing students toward older persons in Ghana were compared. Additionally, the association between the overall quality of students’ experiences with older persons and their attitudes was examined. Materials and Methods: A sample of 135 final year medical and nursing students from a public institution in Ghana participated in a cross-sectional study by completing a web-based self-administered questionnaire consisting of the 14-item University of California at Los Angeles Geriatric Attitudes (UCLA-GA) scale, and demographic questions. Data analysis involved a two-sample t-test and a one-way ANOVA. Results: Most participants (82.2%) held positive attitudes towards older persons. The mean score for the UCLA-GA scale that assessed attitudes of students towards older persons was 3.41 ± 0.41 (min: 2.29, max: 4.64); differences in attitudes between the two groups was significant (p = 0.001). Medical students had more positive attitudes toward older persons than nursing students. The association between students’ attitudes and the overall quality of their experiences with older persons was significant (p = .001). Students whose experiences with older persons were negative had the least positive attitudes. Conclusion: Considering the impact negative experiences with older persons may have on students’ attitudes, attention should be given to creating positive experiences through clinical and community-based exposures. Direct interactions with older persons who are active and living independently may challenge some common stereotypes such as older persons are infirm and senile, and foster positive attitude development.

